# Differential Effects of Ascites and Hepatic Encephalopathy on Waitlist Mortality in Liver Transplantation by MELD 3.0

**DOI:** 10.1097/TXD.0000000000001625

**Published:** 2024-05-15

**Authors:** Brian T. Lee, Nathan T. Chen, Tse-Ling Fong, Jennifer L. Dodge

**Affiliations:** 1 Liver Program, Hoag Digestive Health Institute, Hoag Memorial Hospital Presbyterian, Newport Beach, CA.; 2 Division of Gastrointestinal and Liver Diseases, University of Southern California Keck School of Medicine, Los Angeles, CA.; 3 Department of Population and Public Health Sciences, University of Southern California Keck School of Medicine, Los Angeles, CA.

## Abstract

**Background.:**

MELD 3.0 introduces changes to address waitlist disparities for liver transplant (LT) candidates. Ascites and hepatic encephalopathy (HE) are important milestones in the natural history of cirrhosis regardless of the Model for End-Stage Liver Disease (MELD) score. We aim to assess the impact of ascites and HE and its interaction with MELD 3.0 on waitlist mortality.

**Methods.:**

This is a retrospective study of patients listed for LT in the Organ Procurement and Transplantation Network database from 2016 to 2021. The primary outcome was waitlist mortality (death/delisting for too sick to LT). Ascites/HE were classified as moderate ascites without moderate HE (mAscites), moderate HE without moderate ascites (mHE), both moderate ascites/HE (mBoth), and neither. MELD 3.0 scores were categorized as <20, 20–29, 30–39, and ≥40.

**Results.:**

Of 39 025 candidates, 29% had mAscites, 3% mHE, and 8% mBoth. One-year waitlist mortality was 30%, 38%, and 47%, respectively, compared with 17% (all *P* < 0.001) for those with neither. In multivariable Cox regression, the adjusted risk of waitlist mortality associated with mAscites (versus neither) was a hazard ratio (HR) of 1.76 (95% confidence interval [CI], 1.55-2.00) when the MELD 3.0 score was <20, significantly higher than when the MELD 3.0 score was 20–29 (HR 1.40; 95% CI, 1.27-1.54), 30–39 (HR 1.19; 95% CI, 1.04-1.35), and ≥40 (HR 1.14; 95% CI, 0.91-1.43, interaction *P* < 0.05 for all). A similar pattern was observed by MELD 3.0 for both moderate ascites/HE.

**Conclusions.:**

The presence of moderate ascites alone, or combined with moderate HE, not only increases the risk of waitlist mortality but also has a differential effect by MELD 3.0, especially at lower MELD scores. Earlier strategies addressing this group and improving treatment plans or access to LT regardless of MELD remain needed.

Since 2002, prioritization for organ allocation in liver transplantation (LT) by the United Network for Organ Sharing (UNOS) has relied on the Model for End-Stage Liver Disease (MELD) score of waitlist candidates.^1^ As the experience with LT and waitlist outcomes evolved, disparities in both geographic distribution and prognostic scoring were observed, resulting in several policy changes over time.^2^ Along with revisions in organ distribution policies, the MELD score was modified to include serum sodium, improving prognostication of waitlist mortality for LT in candidates.^3^ However, persistent disparities, especially sex-based, resulted in the latest iteration, referred to as the MELD 3.0, with the inclusion of sex and serum albumin in addition to serum bilirubin, sodium, creatinine, and international normalizing ratio (INR) as variables in the calculation.^4^

The Child-Turcotte-Pugh (CTP) score was devised to prognosticate survival after esophageal transection surgery for the management of bleeding varices.^5^ For years, the CTP score was also used as a clinical tool to assess survival in patients with cirrhosis, and until 2002, it was used by UNOS to prioritize organ allocation for LT.^2^ The score encompasses biochemical parameters of serum bilirubin, albumin, and prothrombin time along with clinical parameters of ascites and hepatic encephalopathy (HE). However, criticisms over the subjectivity of grading ascites and HE plagued the scoring system, creating inequities in organ allocation. These concerns led to UNOS abandoning the CTP score in 2002, in favor of the MELD score, a calculation solely derived from the biochemical parameters of LT candidates.^2^

Nevertheless, the onset of the clinical decompensating events, ascites and HE, mark critical milestones in the natural history of patients with cirrhosis, and they are associated with significantly shorter survival without LT.^6,7^ The onset of minimal HE is associated with shortened 5-y survival^8^ whereas severe HE has a reported 1-y mortality rate of 54%.^9^ The development of ascites in patients with cirrhosis indicates a poor prognosis with an expected 2-y mortality rate of 40%.^10^ In previous iterations of the MELD score, the presence of ascites^11^ or HE^12^ was associated with a higher mortality rate compared with patients with comparable MELD scores without these complications. Gadiparthi et al^13^ evaluated the impact of severe HE on waitlist outcomes in patients with a MELD score of ≥30 patients and found a 58% increased risk of mortality compared with those without HE.

We hypothesize that decompensating events may have a greater impact on patients listed for LT at lower MELD 3.0 scores. Thus, this study aims to evaluate whether the association between waitlist mortality and the clinical decompensating events of ascites and HE differs by levels of MELD 3.0 among LT candidates in the United States.

## MATERIALS AND METHODS

This retrospective cohort study included adults first listed for a primary LT between January 15, 2016, and December 31, 2021, in the Organ Procurement and Transplantation Network (OPTN) database (released December 2022). We excluded candidates listed as Status 1 or with acute hepatic necrosis, listed for non–kidney/liver multiorgan transplant, diagnosed with metabolic disease (tyrosinemia, hyperoxaluria, and maple syrup urine disease), receiving any MELD exception (hepatocellular carcinoma and others), or receiving a living donor LT. Because our study was centered around MELD 3.0 interactions with ascites and HE, listings with missing MELD 3.0 components, ascites, or HE were excluded. Patients listed with a transjugular intrahepatic portosystemic shunt procedure were also excluded because of its effect on reducing ascites and possible complications including an increase in HE.

Information encompassing demographic characteristics, insurance, body mass index (BMI), cause of liver disease, and history of diabetes as well as dialysis, ascites, HE, CTP score, and laboratory data, including serum albumin, bilirubin, creatinine, sodium, and international normalized ratio, were collected at the time of listing. MELD 3.0 was calculated and uncapped for the purposes of this study to more accurately assess the true mortality of patients with MELD 3.0 scores of ≥40,^4^ and patients were sorted into the appropriate MELD 3.0 category at listing: <20, 20–29, 30–39, and ≥40. Individuals were stratified by the presence of moderate ascites and moderate HE (defined as grades 3–4) as neither (absence of moderate ascites and moderate HE), moderate ascites without moderate HE (mAscites), moderate HE without moderate ascites (mHE), or both moderate ascites and moderate HE (mBoth); the moderate classification was used because of the subjective nature of grading diagnoses of ascites and HE. With regards to BMI, individuals were classified dichotomously as underweight (BMI <18.5 kg/m^2^) or not, due to possible BMI inflation from ascites and the higher likelihood of sarcopenia in the former group.

### Outcomes

The primary outcome was waitlist mortality, including both patients dying on the waitlist or being removed for being too sick to undergo transplantation. Patients were followed from the date of listing until mortality with patients censored at the time of LT, delisting for other reasons, or within 1 y of listing for transplant.

### Statistical Analysis

Demographic and clinical characteristics were summarized and stratified by mAscites and mHE groupings, using frequencies with percentages and medians with interquartile ranges, respectively.

The Kaplan-Meier method was used to estimate the 1-y cumulative incidence of waitlist mortality after listing for LT. The log-rank test with Sidak-adjusted *P* values for multiple comparisons was used to quantitatively evaluate differences between the mAscites/mHE groups overall and within MELD 3.0 categories.

Using Cox proportional hazards regression, the independent association between the risk of waitlist mortality and mAscites/mHE groups was quantified as hazard ratios (HRs) with 95% confidence intervals (CIs). The primary model was adjusted for the following covariates selected a priori: MELD 3.0, insurance, blood type, dialysis, diabetes, age, and underweight BMI, and a secondary model was further adjusted for each region. We tested interactions between MELD 3.0 and mAscites/mHE groups to determine whether the effect of these decompensating events differed by MELD 3.0. The multicollinearity condition was checked and satisfied with variance inflation factors <3. Cox regression was selected for the primary model as our focus was on the biologic effects of ascites and HE rather than risk prediction and organ allocation.^14^ However, we used Fine and Gray competing risks regression in a sensitivity analysis to model LT as a competing risk and evaluate MELD 3.0 by mAscites/mHE interactions.

Statistical analyses were conducted in SAS version 9.4 (SAS Institute Inc, Cary, NC). This study was reviewed and deemed exempt by the Institutional Review Board at the University of Southern California.

## RESULTS

### Demographics of Ascites and HE Groups

This study included 39 025 patients listed for LT. The median age at listing was 56 y with 40% women, 73% White, and alcohol (44%) and metabolic dysfunction-associated steatohepatitis (24%) as the predominant causes of liver disease. Decompensating events were present at listing in most patients (87% with any ascites and 71% with any HE), although the more severe manifestations were less frequent with 29% mAscites, 3% mHE, and 8% mBoth. Alcohol as a diagnosis increased with the presence of moderate decompensating events (40% of neither, 48% of mAscites, 47% of mHE, and 57% of mBoth), as did dialysis (9% of neither, 25% of mAscites, 20% of mHE, 28% of mBoth). Median MELD 3.0 scores at listing were highest in mBoth, followed by mHE, mAscites, and those with neither (32 versus 28 versus 25 versus 20, respectively). The MELD components of total bilirubin and INR followed a similar pattern (Table 1). Slight ascites were present in 78.6% and mild HE (grades 1–2) was present in 64.1% of those with neither moderate ascites/HE. Mild HE was present in 74% of mAscites. The mHE group had slight ascites in 85% of patients (Table 1).

**TABLE 1.** Demographics of candidates on UNOS waitlist for liver transplantation

		Ascites and hepatic encephalopathy groups			
Characteristic at listing	Total (N = 39 025)	Neither moderate ascites/HE (N = 23 395)	Moderate ascites (N = 11 324)	Moderate HE (N = 1136)	Both moderate ascites/HE (N = 2970)
Age, y	56 (47–62)	56 (47–62)	56 (48–63)	56 (46–63)	55 (47–61)
Female, n, (%)	15 678 (40)	9754 (41)	4202 (37)	532 (47)	1190 (40)
Ethnicity, n (%)					
White	28 349 (73)	16,952 (72)	8365 (74)	836 (74)	2196 (74)
Hispanic	6329 (16)	3815 (16)	1883 (17)	148 (13)	483 (16)
African American	2571 (7)	1679 (7)	633 (6)	82 (7)	177 (6)
Asian	1097 (3)	752 (3)	259 (2)	37 (3)	49 (2)
Other	679 (2)	397 (2)	184 (2)	33 (3)	65 (2)
Diagnosis, n (%)					
Alcohol	17 190 (44)	9497 (40)	5471 (48)	532 (47)	1690 (57)
MASH	9171 (24)	5487 (23)	2854 (25)	262 (23)	568 (19)
Hepatitis C	3922 (10)	2431 (10)	1103 (10)	99 (9)	289 (10)
Hepatitis B	555 (1)	352 (2)	148 (1)	27 (2)	28 (1)
AILD	4201 (11)	3212 (14)	740 (7)	88 (8)	161 (5)
Other	3986 (10)	2616 (11)	1008 (9)	128 (11)	234 (8)
Body mass index	28.5 (24.7–33.1)	28.5 (24.7–33.1)	28.5 (24.7–33.1)	28.7 (24.7–33.2)	28.8 (24.8–33.4)
Categories, n (%)					
≥30	15 878 (41)	9618 (41)	4534 (40)	473 (42)	1253 (42)
25–29.9	12 706 (33)	7643 (32)	3777 (33)	360 (32)	926 (31)
18.5–24.9	9812 (25)	5955 (25)	2848 (25)	280 (25)	729 (25)
<18.5	553 (1)	338 (1)	145 (1)	17 (2)	53 (2)
Diabetes, n (%)	10 670 (27)	6353 (27)	3241 (29)	313 (28)	763 (26)
Insurance, n (%)					
Private	20 365 (52)	12 555 (53)	5771 (51)	570 (50)	1469 (50)
Medicare	9047 (23)	5557 (24)	2594 (23)	302 (27)	594 (20)
Medicaid	7832 (20)	4435 (19)	2447 (22)	220 (19)	730 (25)
Other	1781 (5)	1048 (4)	512 (5)	44 (4)	177 (6)
Albumin, g/dL	3.1 (2.7–3.5)	3.1 (2.7–3.6)	3.1 (2.7–3.5)	3.0 (2.6–3.5)	3.2 (2.7–3.6)
Bilirubin, mg/dL	3.8 (1.9–9.6)	3.4 (1.8–7.8)	3.9 (1.9–10.1)	6.9 (2.7–20.6)	8.3 (3.3–21.1)
Creatinine, mg/dL	1.10 (0.80–1.79)	1.00 (0.77–1.49)	1.31 (0.90–2.20)	1.21 (0.86–1.91)	1.58 (1.00–2.70)
Dialysis,^*a*^ (n (%)	4914 (13)	2157 (9)	1699 (15)	229 (20)	829 (28)
INR	1.6 (1.3–2.1)	1.5 (1.3–1.9)	1.7 (1.4–2.2)	1.9 (1.5–2.7)	2.1 (1.6–2.7)
Sodium, mmol/L	136 (132–138)	136 (133–139)	134 (131–137)	137 (133–140)	135 (131–139)
MELD 3.0	22 (17–30)	20 (16–27)	25 (19–32)	28 (20–38)	32 (25–39)
MELD 3.0 change from listing to end	+1 (–1 to +5)	+1 (–1 to +5)	+1 (–1 to +5)	+1 (–1 to +4)	+1 (–1 to +4)
MELD 3.0 increase from listing to end, n (%)	20 973 (54)	12 781 (54)	6075 (54)	603 (53)	1,514 (51)
Ascites, n (%)					
Absent	5211 (13)	5044 (21)	0 (0)	167 (15)	0 (0)
Slight	19 520 (50)	18 551 (79)	0 (0)	969 (85)	0 (0.0)
Moderate	14 294 (37)	0 (0)	11 324 (100)	0 (0.0)	2970 (100)
HE, n (%)					
None	11,408 (29)	8459 (36)	2949 (26)	0 (0.0)	0 (0.0)
Grade 1–2	23 511 (60)	15 136 (64)	8375 (74)	0 (0.0)	0 (0.0)
Grade 3–4	4106 (11)	0 (0)	0 (0)	1136 (100)	2970 (100)
CPT score	10 (9–12)	9 (8–11)	11 (9–12)	12 (10–13)	13 (12–14)
Classes, n (%)					
A	1977 (5)	1977 (8)	0 (0)	0 (0)	0 (0)
B	13 714 (35)	10 575 (45)	2899 (26)	165 (15)	75 (3)
C	23 334 (60)	11 043 (47)	8425 (74)	971 (86)	2895 (98)
Follow-up time from listing, mo	2.5 (0.4–12.0)	4.7 (0.7–12.0)	1.3 (0.3–6.7)	0.6 (0.2–4.5)	0.4 (0.1–1.7)

Values are presented as median (IQR) or total N with group proportion as appropriate.

^*a*^Dialysis twice in the past week before listing.

AILD, autoimmune liver disease (autoimmune hepatitis, primary biliary cholangitis, and primary sclerosing cholangitis); CTP, Child-Turcotte-Pugh; HE, hepatic encephalopathy; INR, international normalized ratio; IQR, interquartile range; MASH, metabolic dysfunction-associated steatohepatitis; MELD, Model for End-Stage Liver Disease; UNOS, United Network of Organ Sharing.

The mBoth group had the highest median CTP score, with 98% classified as Child C. Most patients in mHE had Child C (86%) as were patients in mAscites (74%). Those with neither moderate ascites nor HE were often Child classes B or C (45% and 47%, respectively).

### One-year Waitlist Mortality After Listing for LT

**FIGURE 1.**
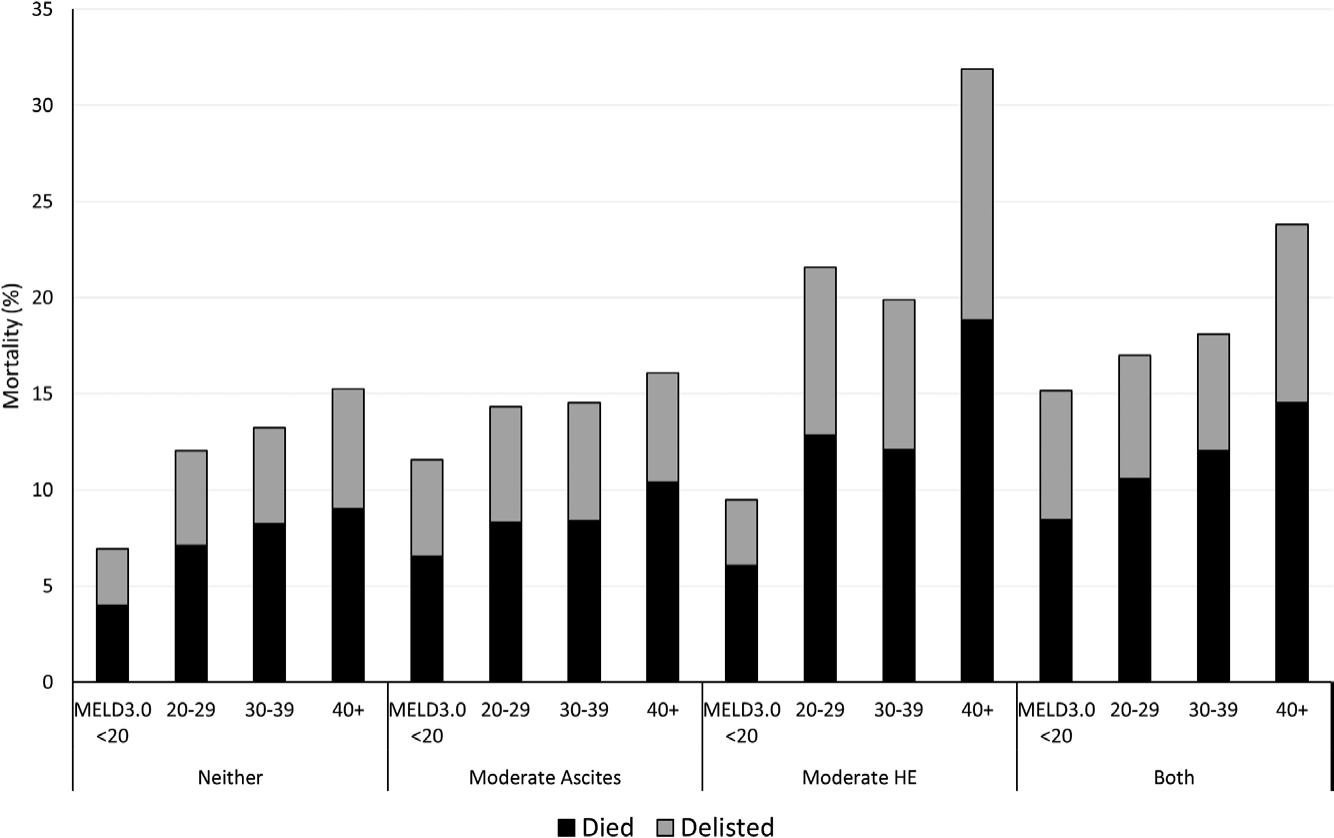
The contribution of deaths and delisting to mortality within 1 y of listing for liver transplant. Waitlist mortality was the composite outcome of death and delisting for being too sick for transplant. The percentage of each mortality component is shown as a stacked bar within categories of moderate ascites/HE and MELD 3.0. HE, hepatic encephalopathy; MELD, Model for End-Stage Liver Disease.

**FIGURE 2.**
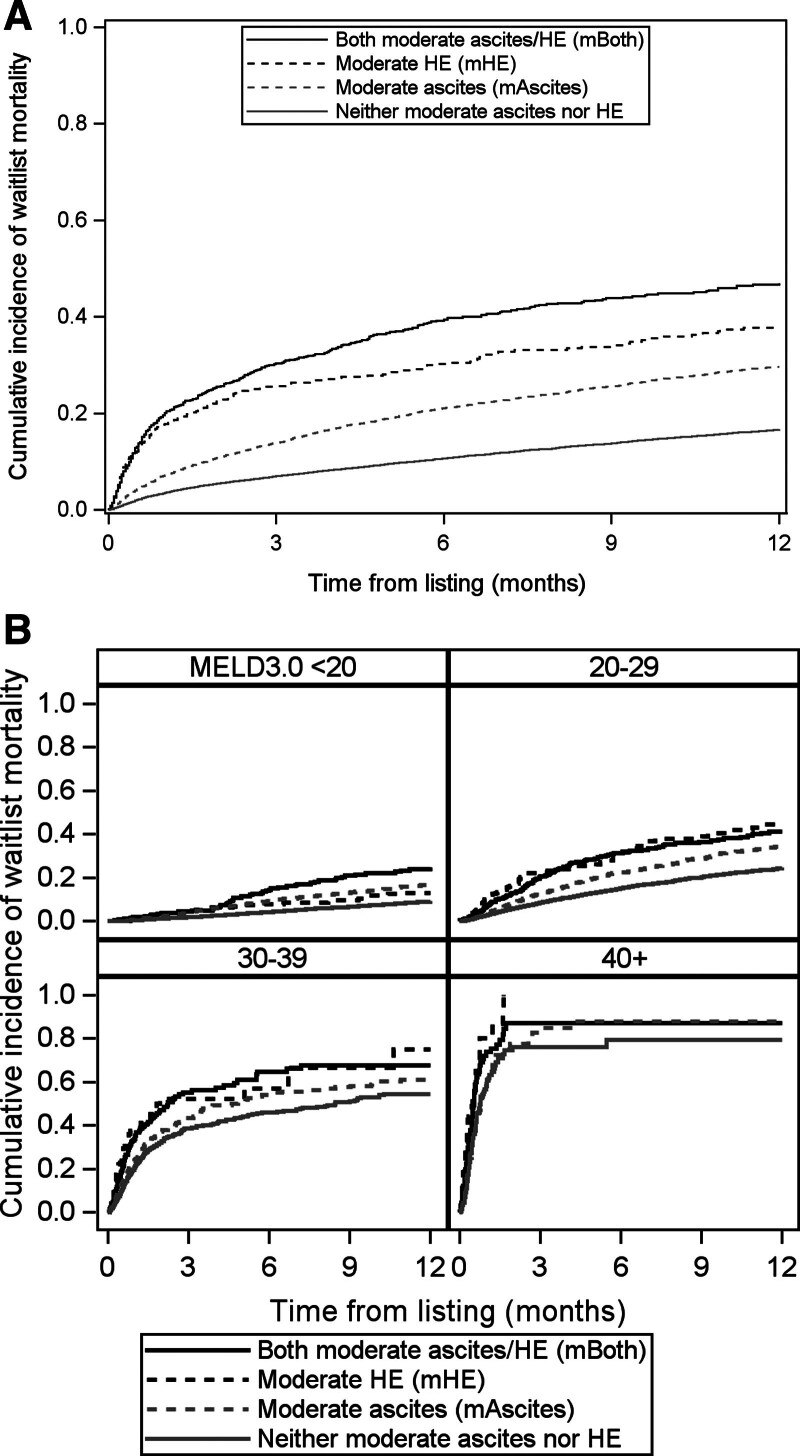
Cumulative incidence of waitlist mortality stratified by the presence of moderate ascites and HE. Mortality was higher for those with moderate ascites, moderate HE, or both present compared with neither (A) in the overall population (all *P* < 0.001) and (B) among those with MELD 3.0 < 20 (all *P* < 0.001), 20–29 (all *P* < 0.001), 30–39 (all *P* < 0.01), and ≥40 for moderate HE and both moderate ascites/HE (all *P* < 0.001). HE, hepatic encephalopathy; MELD, Model for End-Stage Liver Disease.

Within 1 y, 7% of patients died and 5% were delisted for being too sick for LT (**Table S1, SDC,** http://links.lww.com/TXD/A659). The distribution of these mortality events across categories of ascites/HE and MELD 3.0 is shown in Figure 1. The cumulative incidence of waitlist mortality within 1 y was 47% (95% CI, 44-50) for mBoth, 38% (95% CI, 34-42) for mHE, and 30% (95% CI, 28-31) for mAscites compared with 17% (95% CI, 16-17, all *P* < 0.001) for those with neither (Figure 2A).

The cumulative incidence curves for waitlist mortality across MELD 3.0 categories are shown in Figure 2B. In the MELD <20 category, 1-y waitlist mortality was 25% (95% CI, 19-31) for mBoth, 13% (95% CI, 9-19) for mHE, and 17% (95% CI, 15-18) for mAscites compared with 9% (95% CI, 8-10, all *P* < 0.001) for neither. In the MELD 20–29 category, 1-y waitlist mortality was 41% (95% CI, 36-48) for mBoth, 45% (95% CI, 36-54) for mHE, and 35% (95% CI, 32-37) for mAscites compared with 24% (95% CI, 23-26; all *P* < 0.001) for neither. In the MELD 30–39 category, 1-y waitlist mortality was 68% (95% CI, 59-76) for mBoth, 75% (95% CI, 56-91) for mHE, and 61% (95% CI, 56-67) for mAscites compared with 54% (95% CI, 50-59; *P* < 0.01) for neither. In the MELD ≥40 category, 1-y waitlist mortality was 87% (95% CI, 77-94) for mBoth, 100% for mHE, and 88% (95% CI, 44-96) for mAscites compared with 79% (95% CI, 69-88; *P* < 0.001, *P* < 0.001, *P* = 0.90) for neither.

### Risk of Waitlist Mortality

In Cox regression evaluating the moderate ascites/HE groups, the unadjusted HRs for risk of waitlist mortality were 4.48 (95% CI, 4.08-4.92), 3.43 (95% CI, 2.99-3.93), and 1.96 (95% CI, 1.84-2.09) for mBoth, mHE, and mAscites, respectively, compared with neither (*P* < 0.001 for all). After adjusting for age, insurance, blood type, underweight BMI, dialysis, diabetes, and MELD 3.0 categories, the risk of waitlist mortality remained significantly elevated with mAscites (HR 1.40; 95% CI, 1.31-1.49; *P* < 0.001) and mHE (HR 2.20; 95% CI, 1.92-2.53; *P* < 0.001) individually and mBoth (HR 2.02; 95% CI, 1.83-2.23; *P* < 0.001). The adjusted risk of waitlist mortality sequentially increased with higher MELD 3.0 categories (Table 2).

**TABLE 2.** Multivariable Cox regression analysis for mortality within 12 mo of listing for liver transplantation

	Multivariable Cox regression		
Characteristic at listing	Hazard ratio	95% CI	*P*
Age, y			
18–39	1.00		
40–49	1.65	1.43-1.90	<0.001
50–59	2.32	2.04-2.64	<0.001
60–69	3.44	3.02-3.92	<0.001
>69	5.50	4.67-6.48	<0.001
Insurance			
Private	1.00		
Medicaid	1.18	1.09-1.28	<0.001
Medicare	1.25	1.16-1.35	<0.001
Other	1.04	0.89-1.20	0.64
ABO group			
O	1.00		
A	0.99	0.93-1.05	0.65
AB	1.08	0.90-1.29	0.41
B	0.98	0.89-1.09	0.63
Underweight			
BMI <18.5 vs ≥18.5	1.78	1.47-2.15	<0.001
Dialysis	1.08	0.99-1.17	0.07
Diabetes	1.14	1.07-1.22	<0.001
HE/ascites group			
Neither	1.00		
Moderate ascites	1.40	1.31-1.49	<0.001
Moderate HE	2.20	1.92-2.53	<0.001
Both	2.02	1.83-2.23	<0.001
MELD 3.0			
Category 1: <20	1.00		
Category 2: 20–29	2.99	2.78-3.23	<0.001
Category 3: 30–39	15.53	14.16-17.02	<0.001
Category 4: ≥40	65.51	57.48-74.66	<0.001

Multivariable model adjusted for variables listed in table.

CI, confidence interval; HE, hepatic encephalopathy; MELD, Model for End-Stage Liver Disease.

### MELD 3.0 Category Interactions With Ascites/HE

**FIGURE 3.**
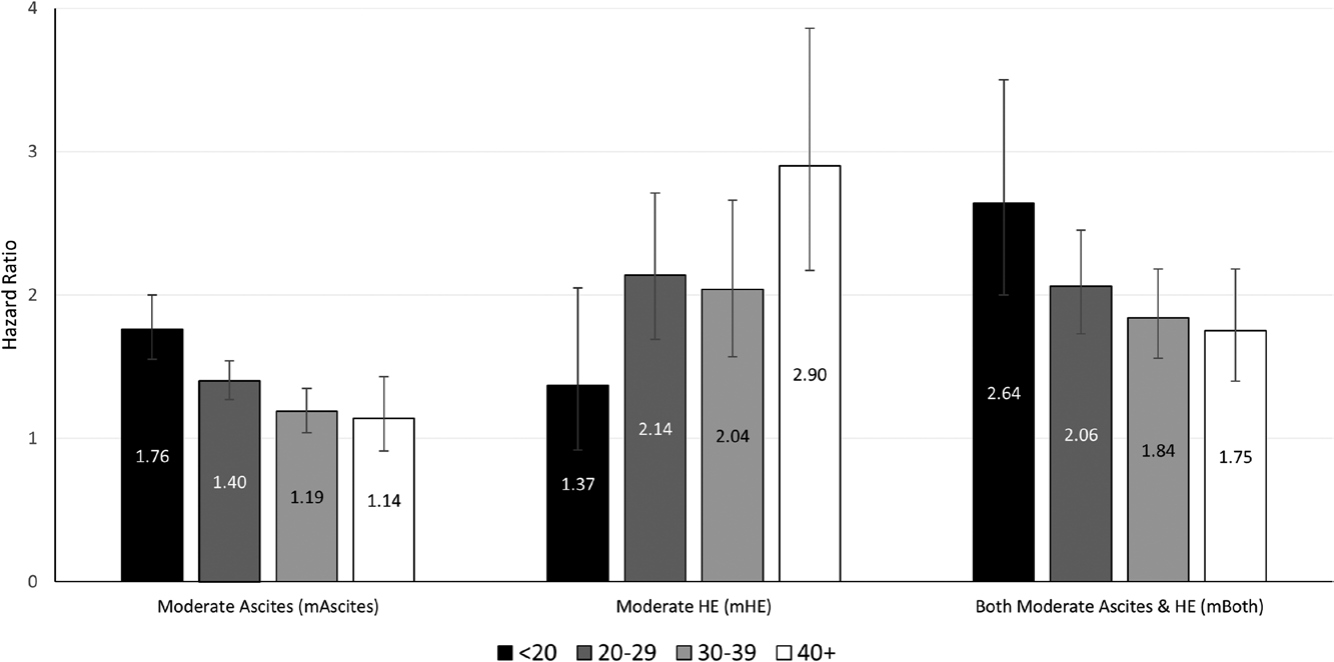
Risk of waitlist mortality associated with the presence of moderate ascites, HE, and both decompensating events by MELD 3.0 category. Hazard ratios^*a*^ are relative to the absence of moderate ascites and moderate HE. The effect of moderate ascites is greatest among patients with MELD 3.0 < 20 and significantly higher than observed in MELD 3.0 categories 20–29 (interaction *P* = 0.005), 30–39 (interaction *P* < 0.001), and ≥40 (interaction *P* = 0.001). The effect of moderate HE was greater at higher MELD 3.0 categories. Similar to moderate ascites, the joint effect of both moderate ascites and HE was greatest in low MELD 3.0 categories compared with MELD 30–39 (interaction *P* = 0.03) and MELD ≥40 (interaction *P* = 0.02). HE, hepatic encephalopathy; MELD, Model for End-Stage Liver Disease. ^*a*^Hazard ratios are adjusted for age at listing, insurance, blood type, underweight status, dialysis, and diabetes.

Within the multivariable Cox regression model, we subsequently assessed for an interaction between the moderate ascites/HE groups and MELD 3.0 categories. The presence of mAscites had a differential effect across MELD 3.0 score categories, with mAscites significantly increasing the risk of waitlist mortality among those with MELD 3.0 scores <20, 20–29, and 30–39 but not achieving statistical significance in the MELD 3.0 ≥40 group (**Table S2, SDC,** http://links.lww.com/TXD/A659). Specifically, the adjusted risk of waitlist mortality for mAscites versus neither was HR 1.76 (95% CI, 1.55-2.00) when the MELD 3.0 score was <20, significantly higher than observed when the MELD 3.0 score was 20–29 (HR 1.40; 95% CI, 1.27-1.54; *P* = 0.005), 30–39 (HR 1.19; 95% 1.04-1.35; *P* < 0.001), and ≥40 (HR 1.14; 95% CI, 0.91-1.43; *P* = 0.001; Figure 3).

For the presence of both moderate ascites/HE, mortality risk was significantly increased compared with those with neither in all MELD subcategories (**Table S2, SDC,** http://links.lww.com/TXD/A659). Notably, the adjusted HR for mBoth versus neither was higher for those with the MELD 3.0 score <20 (HR 2.64; 95% CI, 2.00-3.50) compared with the effect in those with the MELD 3.0 score 20–29 (HR 2.06; 95% CI, 1.73-2.45; *P* = 0.14), 30–39 (HR 1.84; 95% CI, 1.56-2.18; *P* = 0.03), and ≥40 (HR 1.75; 95% CI, 1.40-2.18; *P* = 0.02) with the latter 2 achieving statistical significance (Figure 3).

The pattern of risk associated with mHE by the MELD 3.0 category diverged from that observed with the presence of mAscites. The risk of waitlist mortality for mHE was increased in MELD 3.0 categories >20 but not statistically significant in the low MELD 3.0 score of <20 category (**Table S2, SDC,** http://links.lww.com/TXD/A659). In contrast to the impact of mAscites, there was a higher risk of mortality for mHE versus neither when the MELD 3.0 score was ≥40 (HR 2.90; 95% CI, 2.17-3.86) compared with those with the MELD 3.0 score <20 (HR 1.37; 95% CI, 0.92-2.05; *P* = 0.02; Figure 3).

A secondary model adjusting for the region did not meaningfully change the effect size or significance level of the interaction terms (data not shown).

### Sensitivity Analysis

In a sensitivity analysis using competing risks regression, results were similar to the main analysis, although the magnitude of the MELD 3.0 sub-HRs for risk of waitlist mortality was attenuated (**Table S3, SDC,** http://links.lww.com/TXD/A659). Furthermore, the interactions between MELD 3.0 and moderate ascites/HE groups were confirmed; the sub-HRs were somewhat attenuated but largely maintained statistical significance (**Table S2, SDC,** http://links.lww.com/TXD/A659).

## DISCUSSION

The MELD score remains the core prognostication system in determining prioritization for liver organ allocation. In its latest iteration, the MELD 3.0 improves the prediction of waitlist mortality by the additions of female sex and serum albumin level as variables, including bilirubin by sodium and albumin by creatinine interactions, and setting a new upper limit for creatinine at 3.0 mg/dL.^4^ Nevertheless, there are clinical scenarios where the MELD score may not be as reflective of mortality in cirrhosis (eg, compensated cirrhosis with chronic kidney disease^15^ or patients with low MELD scores who have liver-related complications, otherwise known as “sicker than MELD” patients^16^). Our study highlights the impact of clinical decompensating events, namely ascites and HE, on mortality in patients with chronic liver disease waiting for LT. We show that the relative effect of moderate ascites on the risk of waitlist mortality is greater among patients with MELD 3.0 score <20 compared with other MELD 3.0 score categories, especially the MELD 3.0 score of ≥40. When both moderate ascites and HE are present, the differential effect is again observed, with the relative risk of waitlist mortality greater in those with the MELD 3.0 score <20 compared with higher MELD 3.0 score categories of 30–39 and ≥40.

As chronic liver disease progresses with the development of cirrhosis, the presence of abnormal laboratory findings (bilirubin and platelet count) and the clinical manifestations of portal hypertension (splenomegaly and esophageal varices) correlate with survival.^17^ Sentinel events, including ascites, HE, portal hypertensive bleeding, and jaundice, mark the transition from a compensated to decompensated state, which is associated with a shortened life expectancy.^18,19^ Rates of mortality related to ascites and HE in the first 2 y after diagnosis have ranged from 40% to 54%.^11,12^ The clinical impact of the presence of ascites and HE was initially recognized by Child and Turcotte, leading to the inclusion of these events along with serum bilirubin, serum albumin, and nutritional status in prognosticating outcomes of those undergoing portocaval shunt surgery. These were then categorized into a 3-tier classification.^5^ The Child-Turcotte classification was further revised by Pugh by replacing nutritional status with INR along with adjustments to other variables.^20^ The revised CTP score ranges from 5 to 15, with allocation to classes A, B, or C, depending on the final score. Notably, ascites and HE were allocated equal values as a 3-tier variable in the CTP calculation. However, both complications differ considerably in their impairment of patients with decompensated cirrhosis, and this is seen in their effect on patients across the range of MELD 3.0 scores.

Ascites remain the most common decompensating event in patients with cirrhosis with worsening portal hypertension.^21,22^ Additionally, those with a single event of ascites have a higher risk of further decompensating events in patients with cirrhosis.^6^ Progressive ascites can lead to the formation of refractory ascites, necessitating weekly paracenteses, hydrothorax, and/or hyponatremia.^23^ This can lead to increased basal metabolic rate, protein-calorie malnutrition, and ultimately sarcopenia and functional debility.^24-26^ Spontaneous bacterial peritonitis is another complication of ascites with a high risk of associated hepatorenal syndrome and mortality.^23^ Complications of ascites can affect patients with cirrhosis across the spectrum of MELD scores,^27^ but our study shows that the highest relative effect on mortality risk exists in those with low MELD 3.0 score of <20. This effect was still seen after accounting for the competing risk of LT. This group requires improved management strategies, such as optimizing nutrition, transjugular intrahepatic portosystemic shunt placement,^28,29^ or access to living donor LT/extended criteria donor grafts, to help decrease their risk of waitlist mortality.

HE can be a devastating neuropsychiatric complication in patients with both acute and chronic liver failure. It presents in the presence of liver insufficiency, such as acute liver failure, or as a manifestation of portal hypertension, as seen in decompensated cirrhosis.^30^ The burden of HE in cirrhosis has increased over time, with a 30% increase in hospitalizations, greater healthcare costs, and high readmission rates at approximately 27%.^31-33^ Our study cohort with moderate HE had higher MELD 3.0 scores compared with those with moderate ascites and those with neither. The presence of moderate HE was an independent risk factor of waitlist mortality, consistent with the literature.^13,34^ This was particularly evident in MELD 3.0 scores ≥20, with those with MELD scores ≥40 having the highest risk of waitlist mortality. A previous study on acute-on-chronic liver failure (ACLF) showed that the risk for both short and 1-y mortality in those with HE was heavily modulated by liver function, including bilirubin, INR, creatinine, along with age.^34^The cumulative incidence of mortality was highest in the ACLF-HE group followed by ACLF-no HE, no ACLF-HE, no ACLF-no HE.^34^ Although our study did not look at ACLF criteria, the presence of moderate HE had a greater effect on mortality in higher MELD groups, where a higher prevalence of multiorgan failure is expected. However, although our findings for moderate HE contrasted with those in the moderate ascites group, the presence of both moderate ascites and HE showed the highest relative impact on risk of mortality remained in the lowest MELD 3.0 category.

Limitations of our study include the reliance on data collection from subjective evaluations of ascites and HE based on the OPTN data collection protocol. For this reason, we chose to focus on moderate ascites and HE to limit the degree of subjectivity. A previous study has shown the importance of the 3-level scoring over dichotomization of ascites and HE.^35^ Assessments of portal hypertension, such as the utility of liver stiffness measurements or hepatic-venous pressure gradients, were not available because of the nature of the study. A period of 1 y after listing was chosen to assess survival because of an increase in the waiting period until LT.^36^

In conclusion, although MELD 3.0 may offer improvements in addressing disparities in access to LT, patients with cirrhosis who develop moderate ascites and HE remain at elevated risk of waitlist mortality compared with others with similar MELD 3.0 scores without these decompensating events. This differential effect is especially seen in those with MELD 3.0 scores <20 compared with those with higher MELD 3.0 scores. Although we do not propose incorporating ascites and HE into prognostic scoring systems for LT prioritization, clinicians should remain cognizant of the onset of these landmark events in the management of patients with decompensated cirrhosis. Our study identifies the importance of moderate ascites and moderate HE in patients with low MELD 3.0 scores, given their impact on waitlist mortality. Earlier strategies to address this group of patients and improve their access to LT regardless of MELD are still needed.

## Supplementary Material


