# Supplementary material for: Differential Effects of Ascites and Hepatic Encephalopathy on Waitlist Mortality in Liver Transplantation by MELD 3.0
Source: Transplant Direct. 2024 May 15;10(6):e1625. doi: 10.1097/TXD.0000000000001625 (PMC11098197; doi:10.1097/TXD.0000000000001625)
Supplement: Supplementary file 1 [file txd-10-e1625-s001.pdf]

SUPPLEMENTARY MATERIALS

Supplementary Table 1. Waitlist Outcomes of Patients After Listing to 12 Months

**Outcomes,** n, (%)	**Total** *(N=39,025)*	**Neither moderate ascites nor HE** *(N=23,595)*	**Moderate ascites only** *(N=11,324)*	**Moderate HE only** *(N=1,136)*	**Both moderate ascites and HE** *(N=2,970)*
Died/Delisted*	4,714 (12)	2,369 (10)	1,560 (14)	229 (20)	556 (19)
Liver Transplant	21,845 (56)	11,785 (50)	7,249 (64)	713 (63)	2,098 (71)
Censor	12,466 (32)	9,441 (40)	2,515 (22)	194 (17)	316 (11)

*Delisted for being too sick for transplant

HE = Hepatic Encephalopathy

Supplementary Table 2. Hazard ratios and sub-hazards for risk of death within 12 months of listing for liver transplant amongst MELD groups							
		Multivariable Cox Regression			Multivariable Fine and Gray Competing Risk		
**Parameter**	**Ascites/HE**	**Hazard Ratio**	**95% CI**	**p-value Ref MELD <20**	**Hazard Ratio**	**95% CI**	**p-value Ref MELD <20**
Interaction with MELD				<.001			<.001
MELD3.0 Category 1: <20	Neither	1.00			1.00		
	Moderate Ascites	1.76	1.55-2.00		1.57	1.39-1.78	
	Moderate HE	1.37	0.92-2.05		1.24	0.84-1.84	
	Both	2.64	2.00-3.50		2.03	1.55-2.66	
MELD3.0 Category 2: 20-29	Neither	1.00			1.00		
	Moderate Ascites	1.40	1.27-1.54	0.005	1.16	1.05-1.28	0.0001
	Moderate HE	2.14	1.69-2.71	0.06	1.72	1.36-2.18	0.16
	Both	2.06	1.73-2.45	0.14	1.40	1.18-1.66	0.02
MELD3.0 Category 3: 30-39	Neither	1.00			1.00		
	Moderate Ascites	1.19	1.04-1.35	<.001	1.05	0.91-1.20	<.001
	Moderate HE	2.04	1.57-2.66	0.10	1.49	1.13-1.97	0.46
	Both	1.84	1.56-2.18	0.03	1.33	1.12-1.59	0.01
MELD3.0 Category 4: 40+	Neither	1.00			1.00		
	Moderate Ascites	1.14	0.91-1.43	0.001	1.05	0.82-1.33	0.003
	Moderate HE	2.90	2.17-3.86	0.003	2.69	1.93-3.75	0.003
	Both	1.75	1.40-2.18	0.02	1.61	1.27-2.05	0.21

Multivariable model adjusted for variables listed in Table 2

CI = Confidence Interval; HE = Hepatic Encephalopathy; MELD = Model for End-Stage Liver Disease

**Supplementary Table 3. Multivariable Competing Risk Regression for Mortality Within 12 months of Listing for Liver Transplantation**				
		**Multivariable Fine and Gray Competing Risk**		
**Characteristic at listing**		**Sub-Hazard Ratio**	**95% CI**	**p-value**
Age	18-39	1.00		
	40-49	1.53	1.33-1.76	<.001
	50-59	2.06	1.80-2.34	<.001
	60-69	3.08	2.70-3.52	<.001
	>69	4.81	4.07-5.68	<.001
Insurance	Private	1.00		
	Medicaid	1.29	1.19-1.39	<.001
	Medicare	1.25	1.16-1.35	<.001
	Other	1.05	0.90-1.21	0.56
ABO Group	O	1.00		
	A	1.02	0.95-1.08	0.65
	AB	0.63	0.53-0.75	<.001
	B	0.80	0.72-0.88	<.001
Underweight	BMI <18.5 vs ≥18.5	2.05	1.69-2.48	<.001
Dialysis		1.18	1.08-1.28	<.001
Diabetes		1.20	1.13-1.28	<.001
HE/Ascites Group	Neither	1.00		
	Moderate Ascites	1.21	1.13-1.29	<.001
	Moderate HE	1.79	1.55-2.06	<.001
	Both	1.52	1.37-1.68	<.001
MELD 3.0	Category 1: <20	1.00		
	Category 2: 20-29	1.71	1.59-1.84	<.001
	Category 3: 30-39	2.09	1.91-2.28	<.001
	Category 4: >39	2.96	2.62-3.35	<.001

Multivariable model adjusted for variables listed in table

CI = Confidence Interval; HE = Hepatic Encephalopathy; MELD = Model for End-Stage Liver Disease
